# Editorial: Celiac Disease

**DOI:** 10.3389/fped.2020.00328

**Published:** 2020-06-24

**Authors:** Ron Shaoul, Andrew S. Day

**Affiliations:** ^1^Pediatric Gastroenterology & Nutrition Institute, Ruth Children's Hospital of Haifa, Rambam Medical Center, Faculty of Medicine, Technion, Haifa, Israel; ^2^Department of Paediatrics, University of Otago Christchurch, Christchurch, New Zealand

**Keywords:** celiac, pathogenesis, treatment, epidemiology, prevention, manifestations

The manifestations and broad impact of Celiac disease (CD), a chronic inflammatory disorder of the small bowel mediated by immune responses to triggering peptides from dietary gluten, are now increasingly recognized (1). This special issue draws together a series of reports focusing on different aspects of this important pediatric condition.

## Epidemiology of Celiac Disease

The presentation patterns of CD have been observed to have changed and evolved in recent decades. Popp and Maki outline the key changes in the epidemiology of CD and illustrate the likely environmental and other factors that may have derived these changes.

## Pathogenesis

In recent years, there have further advances in our understanding of CD. These include the role of cereals, intra-luminal digestion, epithelial barrier function, tissue transglutaminase enzymatic activity, genetic factors, and immune responses.

In their review, Tye-Din et al. delineate the important aspects of the aetiopathogenesis of CD. Although the emphasis is upon genetic susceptibility and dietary exposure to cereal peptides, this report draws attention to other potential factors including host-pathogen interactions and concurrent environmental factors. As outlined, further advances in understanding these key aspects should assist in advances in diagnosis and management. The authors further emphasize that additional work is required to understand environmental triggers, interactions with the microbiota and raise potential for prevention and enhanced management and even prevention.

Smigoc Schweiger et al. explored the relationship between polymorphisms in genes linked to the inflammasome in CD and in type 1 Diabetes. The researchers evaluated the presence of polymorphisms in selected genes in small groups of Slovenian children with T1D, CD or both conditions. Although they documented a link between the development of T1D and a polymorphism in the protein tyrosine phosphatase non-receptor 22 (PTPN22) gene, this polymorphism was associated with protection from CD. While the mechanism of this protective benefit was not elucidated, this work emphasizes the importance of non-HLA genes in the development of CD. Further attention to this enzyme and it's potential adverse impacts is required.

In another aspect of the pathogenesis of CD, Torsten and Aaron review the potential impact of microbial transglutaminase (TG). While it is well-established that mucosal TG plays an important role in de-amidating cereal peptides, the role of microbial sourced TG has not been fully considered. The authors outline the chemical nature of this enzyme, outline the current industrial roles, and characterize how this enzyme could also impact upon CD.

## Manifestations of CD

Although CD in primarily a condition affecting the small bowel, there may be various and widespread manifestations outside the gut. Nardecchia et al. reviewed the patterns of these extra-intestinal manifestations (EIM) with emphasis upon the particular patterns of these features in children (as distinct to adults). The authors also explored the potential mechanisms for CD-related EIM. Two key mechanisms were detailed: those consequent to intestinal malabsorption and those consequent to immune responses. This work helps to emphasis the nature of these EIM and draws attention to the early recognition of these features in children who may not present with any gastrointestinal symptoms.

## Patient Management

Meijer et al. discuss the potential for prevention of CD. Recent work has shown that early feeding steps may not prevent CD in children at greater genetic risk (2–4) and further work is required in this population. Further, primary, secondary, and tertiary prevention and management strategies are outlined and emphasized. At present, such strategies are not fully understood, and further work is required to answer the outstanding questions. This may enhance outcomes in due course.

In addition, Yoosuf and Makharia highlight the range of novel therapies that are being evaluated and considered as options for the management of CD. None of these therapies are established as valid and effective options, and it is not clear if any of these will be complete alternatives to the GFD. But none-the-less, these options provide optimism for the future.

## Controversies

There are several controversies in the management of CD. Cohen et al. (5) recently reviewed the issue of how much dietary gluten is safe in people with CD. Total daily gluten consumption that seems to be safe for most CD patients is <50 mg gluten, nevertheless, some CD patients need as little as 10 mg of gluten daily to promote development of intestinal mucosal abnormalities. Therefore, they recommend that until new data will be available the recommendation for children should be a GFD with <20 ppm as defined by the Codex.

Two articles explore key controversies in the management of a GFD for CD.

Spector Cohen et al. reviewed the dilemma of including oats in a GFD. It appears that pure oats are well-tolerated by most CD patients at moderate amounts (20–25 gr/day dry rolled oats for children; 50–70 gr/day for adults). Nevertheless, due to existing uncertainty it is suggested that oats should be added with caution to a GFD, only after all CD symptoms including weight loss and growth disturbances have resolved, after at least 6 months of a conventional GFD and only after normalization of serology. Furthermore, these patients should be closely monitored.

While the GFD is currently the only intervention available for individuals with CD, Lerner et al. outline the potential adverse implications of a GFD. This article highlights that a GFD could lead to nutritional deficiencies and mental health outcomes, and emphasizes the importance of dietetic oversight of a GFD.

## Conclusions

Together the articles comprising this special issue provide important and timely updates about the current status of CD in children across the world. Each report raises questions and indicates aspects that require further attention and scientific enquiry. While the understanding of the pathogenesis and manifestations of CD has advanced greatly in recent years, there remains controversy and lack of a cure.

## Author Contributions

All authors listed have made a substantial, direct and intellectual contribution to the work, and approved it for publication.

## Conflict of Interest

The authors declare that the research was conducted in the absence of any commercial or financial relationships that could be construed as a potential conflict of interest.
